# Modelling the effects of weather and climate on malaria distributions in West Africa

**DOI:** 10.1186/1475-2875-13-126

**Published:** 2014-03-28

**Authors:** Ali Arab, Monica C Jackson, Cezar Kongoli

**Affiliations:** 1 Department of Mathematics and Statistics, Georgetown University, Washington, DC 20057, USA; 2 Department of Mathematics and Statistics, American University, Washington, DC 20016, USA; 3 Earth System Science Interdisciplinary Center (ESSIC), University of Maryland, College Park, MD, USA; 4 National Oceanic and Atmospheric Administration (NOAA), National Environmental Satellite Data and Information Service (NESDIS), College Park, MD, USA

## Abstract

**Background:**

Malaria is a leading cause of mortality worldwide. There is currently conflicting data and interpretation on how variability in climate factors affects the incidence of malaria. This study presents a hierarchical Bayesian modelling framework for the analysis of malaria *versus* climate factors in West Africa.

**Methods:**

The hierarchical Bayesian framework takes into account spatiotemporal dependencies, and in this paper is applied to annual malaria and climate data from ten West African countries (Benin, Burkina Faso, Côte d'Ivoire, Gambia, Ghana, Liberia, Mali, Senegal, Sierra Leone, and Togo) during the period 1996-2006.

**Results:**

Results show a statistically significant correspondence between malaria rates and the climate variables considered. The two most important climate factors are found to be average annual temperature and total annual precipitation, and they show negative association with malaria incidence.

**Conclusions:**

This modelling framework provides a useful approach for studying the impact of climate variability on the spread of malaria and may help to resolve some conflicting interpretations in the literature.

## Background

Malaria is a leading cause of infectious disease and death worldwide with 3.3 billion people at risk for contracting the disease. In 2010, an estimated 219 million (range 154 million to 289 million) became infected with malaria, of which an estimated 660,000 people died [1]. Malaria is caused by a single-celled parasite of the genus *Plasmodium*, which is transmitted among humans by female mosquitoes of the genus *Anopheles*. The successful development of the malaria parasite in the mosquito depends on several factors, most importantly on temperature and humidity (higher temperatures accelerate the parasite growth in the mosquito) and whether *Anopheles* survives long enough to allow the parasite to complete its cycle in the mosquito host [2].

The ecology of malaria is complex, with multiple biophysical and socio-economic factors impacting the disease. Studies of the various environmental factors impacting epidemics of malaria in Africa, where the disease is most prevalent, have helped to shed light on how climate variables may affect the vector mosquito population and the parasite it carries. Recent research has focused primarily on temperature and rainfall with somewhat conflicting results.

The role of temperature on malarial epidemics was demonstrated by a retrospective study of malarial cases in the highland region of East Africa from 1970 to 2003 [3]. This study found an association between malaria epidemics and warmer temperatures, although the predicted size of the epidemics was smaller than what actually took place. These results led to the conclusion that factors other than temperature must have also affected the outcome. Chua [4] also found that increased temperature favours the survival of malaria-carrying mosquitoes, but the degree to which a rise in temperature increases the spread of malaria is dependent on the baseline temperature, with cooler regions experiencing the largest change. As such, climate change is most likely to increase the spread of malaria in high altitude areas, particularly those with an altitude over 2,000 m because lower regions are already sufficiently warm for the breeding of the mosquito vector [5]. Parham and Michael [6] found that the endemic transmission of malaria, and the rate at which it spreads in a disease-free region, are optimized at 32-33°C. On either side of this range, fewer mosquitoes survive long enough for the parasite to complete its life cycle within the host. Gillioli and Mariani [7] came to the same conclusion, although they found that the bell-shaped distribution of mosquito population peaked at 24-25°C and fell off with any deviation to either side of that ideal temperature. Mordecai *et al.*[8] did a comprehensive study on the role of temperature on malaria spread using a semi-empirical model and analysing observational data dating back one century. They found that the optimal temperature for malaria transmission peaks at 25°C, much lower than previously thought, i.e. 32-33°C. This study also found that malaria spread decreases dramatically for temperatures above 28°C. The authors note that previous studies that established much higher optimal temperatures are at odds with long-term observational data dating back one century. Recently, Siraj *et al.*[9] analysed spatiotemporal malaria and climate data at a regional scale in highlands of Colombia and Ethiopia to examine how the spatial distribution of the disease changes with the interannual variability of temperature. They found an increase in malaria incidence in warmer years. Also, a recent analysis [10] of future projections of global malaria distributions based on bias-corrected temperature and rainfall simulations from climate models showed (although with large uncertainties) an overall global net increase in the population at risk.

The amount of rainfall is another factor found to impact mosquito populations and the spread of malaria. Using a mathematical model of malaria transmission and performing a sensitivity analysis, Gillioli and Mariani [7] found that mosquito populations display a positive quasi-linear response pattern to rainfall variation, but with less sensitivity to rainfall than to temperatures. Chaves *et al.*[11] also found a positive association between malaria outbreaks and rainfall, probably because it creates many stagnant pools of water, which are fertile breeding grounds for mosquitoes.

The goal of the current study is to present a hierarchical Bayesian statistical modelling framework that can be used to analyse the effect of multiple climate factors on the distribution of malaria while taking into account spatiotemporal dependencies. Several studies [12-15] have used Bayesian spatiotemporal models demonstrating their utility in modelling malaria distribution and its associated environmental and socio-economic factors at local scales. Here, the focus is on the regional-scale modelling of malaria incidence in several West African countries with annual climate factors as covariates. A large-scale analysis of this endemic region is preferred for establishing potential malaria links to inter-annual climate variability.

## Methods

### Data

This study examines reported malaria cases and deaths from ten countries (Benin, Burkina Faso, Côte d'Ivoire, Gambia, Ghana, Liberia, Mali, Senegal, Sierra Leone, and Togo) in West Africa linked with climate data obtained from the National Oceanic and Atmospheric Administration's (NOAA) National Climate Data Center (NCDC) [16] and published in [17].

The reported malaria cases and deaths for an 11-year period (1996-2006) were obtained from [18]. Annual malaria rates were computed as malaria cases divided by the associated population size in each country. Climate data were measured at weather stations available for each country. When more than one weather station was available, the most central location for the geographic index is used, which was also used to measure distances in the spatial analysis. Missing values were imputed using the average values of the available years.

**Figure 1:**
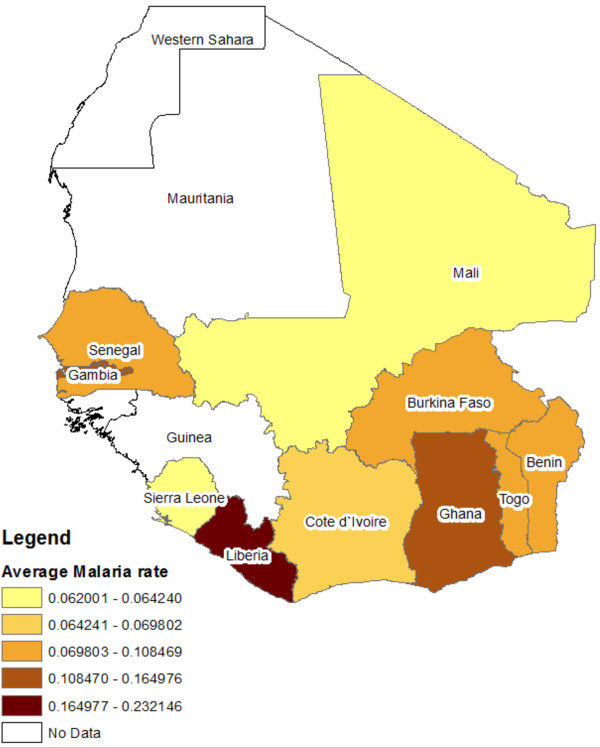
Geographical map of average malaria rates.

**Figure 2:**
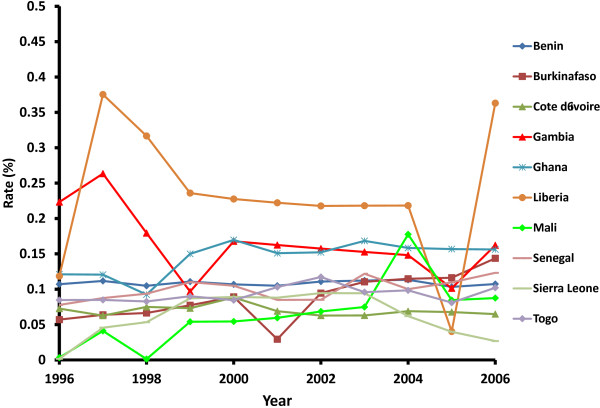
Annual malaria rates for the ten countries selected.

Figure 1 displays a map of mean malaria rates for the ten countries selected, whereas Figure 2 displays time series plots of annual malaria rates. It is shown that Côte d’Ivoire had the lowest and most stable malaria rate with a mean of 0.06%. Interestingly, Mali had a low rate that increased by two orders of magnitude, from a low value of 0.003% in 1996 to 0.06% in 2007, and that peaked in 2004 at a rate of 0.18%. On the other hand, Liberia had the highest mean rates (0.23%) and largest inter-annual variation fluctuating between a maximum value of 0.38% in 1997 and a minimum value of 0.04% in 2005. Overall, most countries had a drop in malaria rates in 2005 and a rise in 1997.

Monthly climate data were obtained for several variables and averaged to obtain annual values to link with the malaria data. The variables used are mean station pressure (mb) denoted by “mstpr”, mean sea level pressure (mb) denoted by “msper”, mean temperature (°C) denoted by “mtmp”, departure of temperature from long term station average (°C) denoted by “dtpav”, mean vapour pressure (mb) denoted by “mvp”, number of days with precipitation at least 1 mm denoted by “dp”, total precipitation (mm) denoted by “totp”, and departure of precipitation from long term station average (mm) denoted by “dpave”. Table 1 displays mean values of the climate variables considered.

**Table 1:** Mean values of the climate variables considered

**Country**	**Mstpr**	**Msepr**	**Mtmp**	**Dtpav**	**Mvp**	**Dp**	**Totp**	**Pop**	**Rate**
Benin	10,002.91	10,109.64	27.42	1.74	291.16	6.20	101.96	6,871,646	0.11
Burkina Faso	9,676.74	10,088.75	28.07	-2.28	192.49	5.96	136.49	12,169,430	0.09
Côte d’Ivoire	10,086.67	10,110.37	27.34	0.84	292.88	7.59	122.54	17,288,436	0.07
The Gambia	10,079.86	9,977.20	27.29	2.80	230.65	5.59	96.65	1,414,390	0.16
Ghana	10,078.94	10,121.23	27.23	0.00	296.77	5.46	71.80	20,227,154	0.15
Liberia	9,780.00	10,089.00	25.90	0.00	274.00	1.00	11.00	2,647,139	0.23
Mali	9,685.94	10,098.72	27.89	0.35	185.21	5.84	89.10	10,345,394	0.06
Senegal	10,102.72	10,124.10	26.53	12.14	237.63	4.08	86.05	11,032,891	0.10
Sierra Leone	9,980.27	10,102.76	28.52	15.16	287.20	5.03	1796.56	5,170,957	0.06
Togo	9,884.56	10,108.77	27.35	2.66	251.75	5.50	112.63	4,851,532	0.09

### Spatiotemporal hierarchical model

The goal is to develop a statistical model that utilizes data on the number of cases of malaria and potential variables that may be correlated with the spatial and/or temporal variability of the number of malaria cases. To this end, a spatiotemporal modelling approach within a hierarchical Bayesian framework is adopted. The spatial aspect of the modelling approach allows for taking into account similarities between values observed at locations that are located closer, ultimately allowing for “borrowing strength” across space. Similarly, the temporal aspect of the modelling approach allows for inference concerning temporal trends of changes of malaria cases. Finally, the proposed model allows for spatial and temporal trend analysis of the data as well as considering the effect of predictor variables.

In particular, for a hierarchical model the following general stages [19] are defined: *data model*, *process model*, and *parameters models*. The data model is given by:

$$z_{t}\sim \mathrm{Poi}\left(\lambda_{t}\right),\;t=1,\ldots ,T,$$

where **z**_t_ is the vector of malaria cases data at time *t*, **z**_*t*_ = (*z*_*t*_(*s*_1_), *z*_*t*_(*s*_2_), …, *z*_*t*_(*s*_*n*_))′ for locations *s*_1_, *s*_2_, …, *s*_*n*_ with number of years, t = 1,…,*T* = 11, and the number of countries in the study, *n* = 10. These data are assumed to follow a Poisson distribution with intensity ***λ***_*t*_ = (*λ*_*t*_(*s*_1_), *λ*_*t*_(*s*_2_), …, *λ*_*t*_(*s*_*n*_))′. The Poisson model uses population (denoted by “Pop”) as an *offset* variable (i.e., intensity is the ratio of mean number of cases and population). Poisson distribution is the common and standard choice for modelling rates. However, other data models may be considered.

The process model is given by:

$$\log\left(\lambda_{t}\right)=\log\left(\mathrm{Po}p_{t}\right)+\beta_{0}+\mu_{1}t+\mu_{2}t^{2}+\sum_{k=1}^{8}\beta_{k}X_{k},$$

where *β*_0_ is the intercept, ***μ***_***1***_ = (*μ*_1_(*s*_1_), *μ*_1_(*s*_2_), …, *μ*_1_(*s*_*n*_))′ is the spatially-varying regression coefficient for time (ie, linear and spatially-varying trend), ***μ***_2_ = (*μ*_2_(*s*_1_), *μ*_2_(*s*_2_), …, *μ*_2_(*s*_*n*_))′ is the spatially-varying regression coefficient for the second-order variable for time (i e, quadratic and spatially-varying trend), *β*_*k*_ denotes the *k*th regression coefficient with *k* = 1,…,8. In this stage of the model, the regression model allows statistical learning from the predictors about the response variable, which is the log of the Poisson intensities. Note that population is used as the offset variable in the Poisson log-linear model. Also, it should be noted that time series models such as autoregressive models on the error terms may be considered in order to address temporal dependence in the data. This modelling strategy was considered in the preliminary analysis of the data. However, the modelling selection procedure significantly favoured models with time as a predictor variable.

A useful aspect of the proposed model is the ability to account for the spatially varying regression coefficients, which facilitate a more flexible and robust interpretation of results over both spatial and temporal scales. The spatial structure is assumed for the linear and quadratic trend coefficients though a covariance structure ( $\Sigma_{\mu_{j}}$) on their prior distribution:

$$\mu_{j}\sim N\left(0,\Sigma_{\mu_{j}}\right),\;j=1,2,$$

$$\Sigma_{\mu_{j}}=\sigma_{\mu_{j}}^{2}R\left(\tau_{j}\right),$$

The spatial correlation is considered based on an exponential covariogram model

$$R\left(\tau_{j}\right)=\exp\left(-\tau_{j}d\right),\;j=1,2,$$

where the spatial correlation is based on the Euclidean distance (***d***) and a spatial range parameter, *τ*_*j*_ (which governs the strength of spatial correlation over spatial locations). A symmetric correlation function is assumed. Many other choices for the spatial correlation function exist including models for areal data (e g, see [20]).

The prior densities described above as well as other prior densities that should be defined for unknown parameters are all part of the third stage of the hierarchical model (parameter models). The prior distributions are defined based on relatively non-informative distributions with small mean and large variances for all the unknown parameters. In particular, the priors for constant regression coefficients are defined as normal distributions with mean 0 and variance 100, and priors for each of the variance components are defined as inverse-Gamma distributions with mean 0.1 and variance 100. The choices of priors considered here are standard choices in the Bayesian modelling literature [21-23], however other reasonable choices of prior distributions may be considered and results should not be sensitive to these choices (note that non-standard choices may require additional computational burden and/or algorithmic strategies).

Ultimately, there is interest in drawing inference about the unknown parameters and the process (ie, intensity parameters) conditioned on data. Using the Bayes’ theorem, the joint posterior distribution of the unknown parameters is proportional to the product of the sampling distribution and the prior densities. Often, the joint posterior is too complex and instead, one can draw samples from it using computational approaches such as Markov chain Monte Carlo (MCMC). MCMC methods are popular for simulating from complex posterior distributions. MCMC methods are a class of algorithms for sampling from probability distributions based on construction of a Markov chain that has the desired distribution as its stationary distribution [21] and includes algorithms such as the Metropolis–Hastings and the Gibbs sampler [22,23]. Gibbs sampling is the main basis of the freely distributed software WinBUGS/OpenBUGS [24], which was used to fit this model.

In order to assess the model goodness-of-fit and selection of the “best” model from the pool of candidate models, several different models were considered and model selection was conducted based on a commonly used method for hierarchical Bayesian models called the deviance information criterion (DIC). DIC introduced by [25] as a generalization of Akaike's information criterion (AIC), is a penalized likelihood method based on the posterior distribution of the deviance statistic defined as

$$D\left(\theta \right)\approx -2\log\left(p\left(y|\theta \right)\right),$$

Where y denotes the data, *p*(*y|θ*) denotes the sampling distribution (i e, likelihood function) of data given model parameters (θ), and *C* is a constant.

DIC is defined as

$$\mathrm{DIC}=2\bar{D}-D\left(\bar{\theta }\right)$$

where $\bar{D}$ is the posterior mean of the deviance, and $D\left(\bar{\theta }\right)$ is the deviance of the posterior mean values for the model parameters (denoted by *θ*). Based on the DIC criterion, models with relatively lower DIC values indicate a better fit to the data compared to models with higher DIC values.

Three different models were considered: a model with spatially varying linear and quadratic trends (M1), a model with spatially varying linear trend and no quadratic trends (M2), and a model with constant linear trend and no quadratic trends (M3). Additional models were also applied to check for multicollinearity in the variables. Mainly, departure of precipitation from long term station average (dpave) was dropped from the models due to collinearity (dpave was negatively correlated with departure from average temperature, dtpave, with correlation coefficient -0.63).

## Results

Results were obtained based on 90,000 MCMC realizations after ignoring the first 10,000 as the “burn-in” period. Based on DIC values, the model with spatially varying linear and quadratic trends (M1) performed best among the models considered (DIC = 1320), followed by the model with spatially varying linear trend, M2 (DIC = 1454). The model without the spatially varying trend term, M3 had the worst performance (DIC = 1985). Based on these results, it is clear that including spatially varying trend terms is necessary.

**Figure 3:**
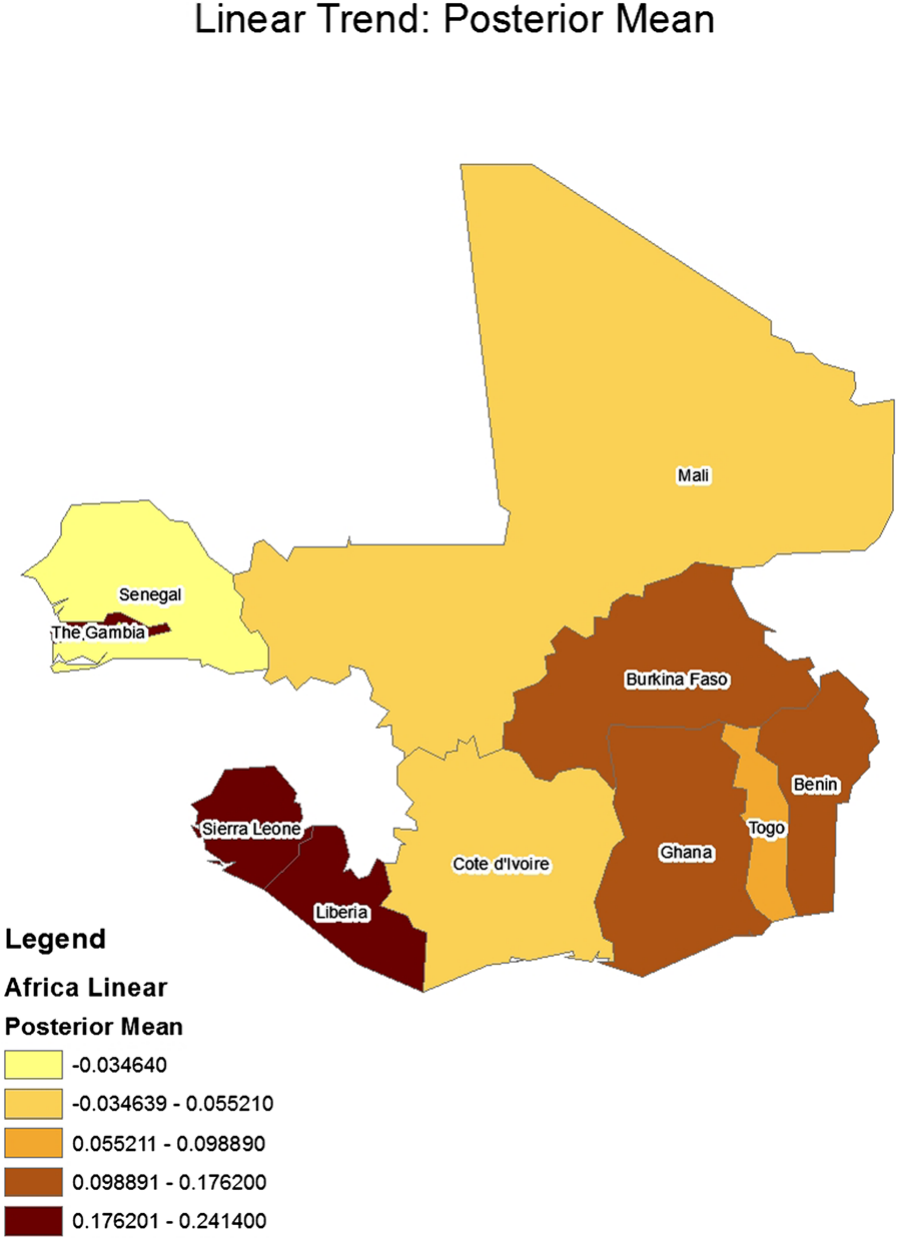
Geographical map of posterior mean results for the spatially-varying linear trend coefficient.

**Figure 4:**
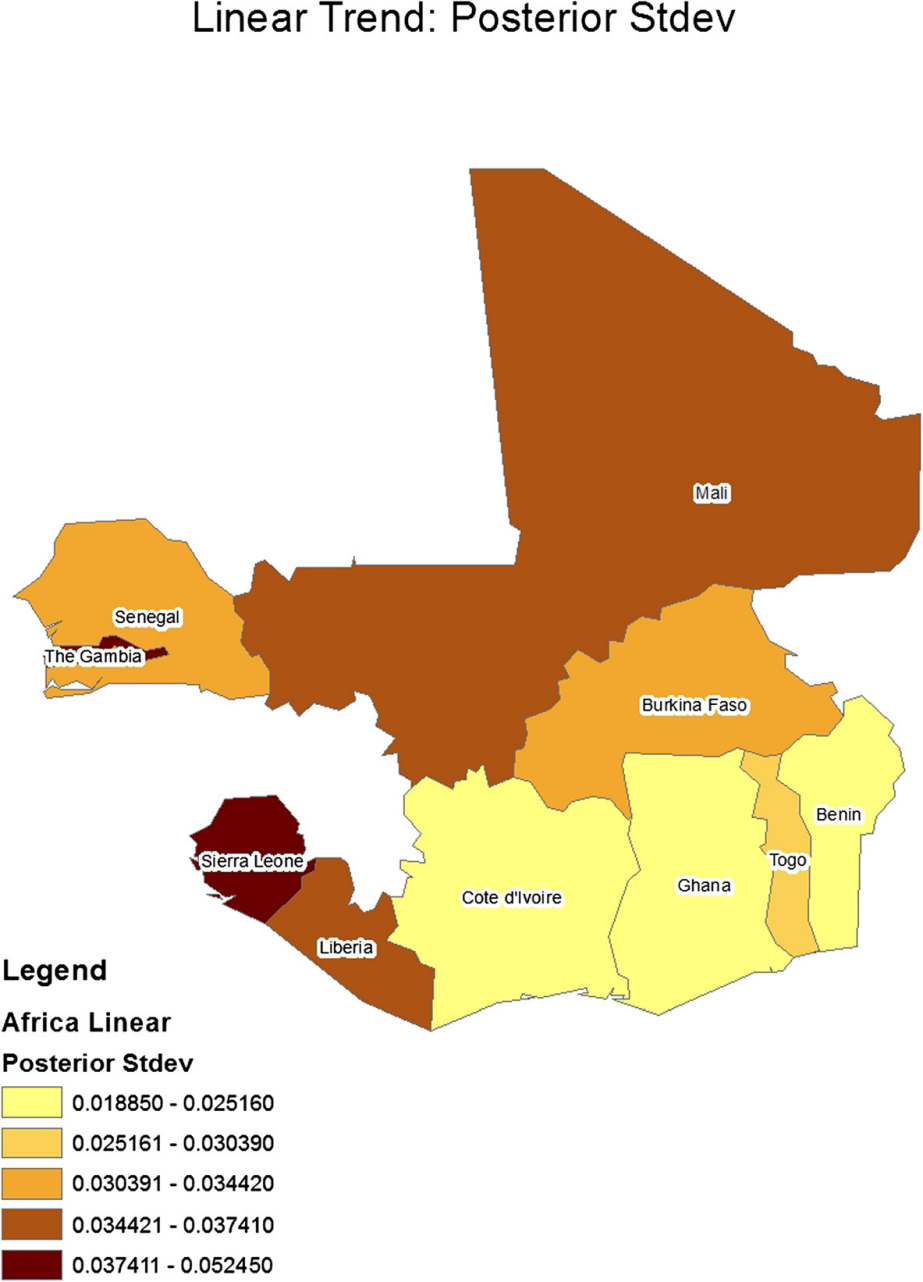
Geographical map of posterior standard deviation results for the spatially-varying linear trend coefficient.

**Figure 5:**
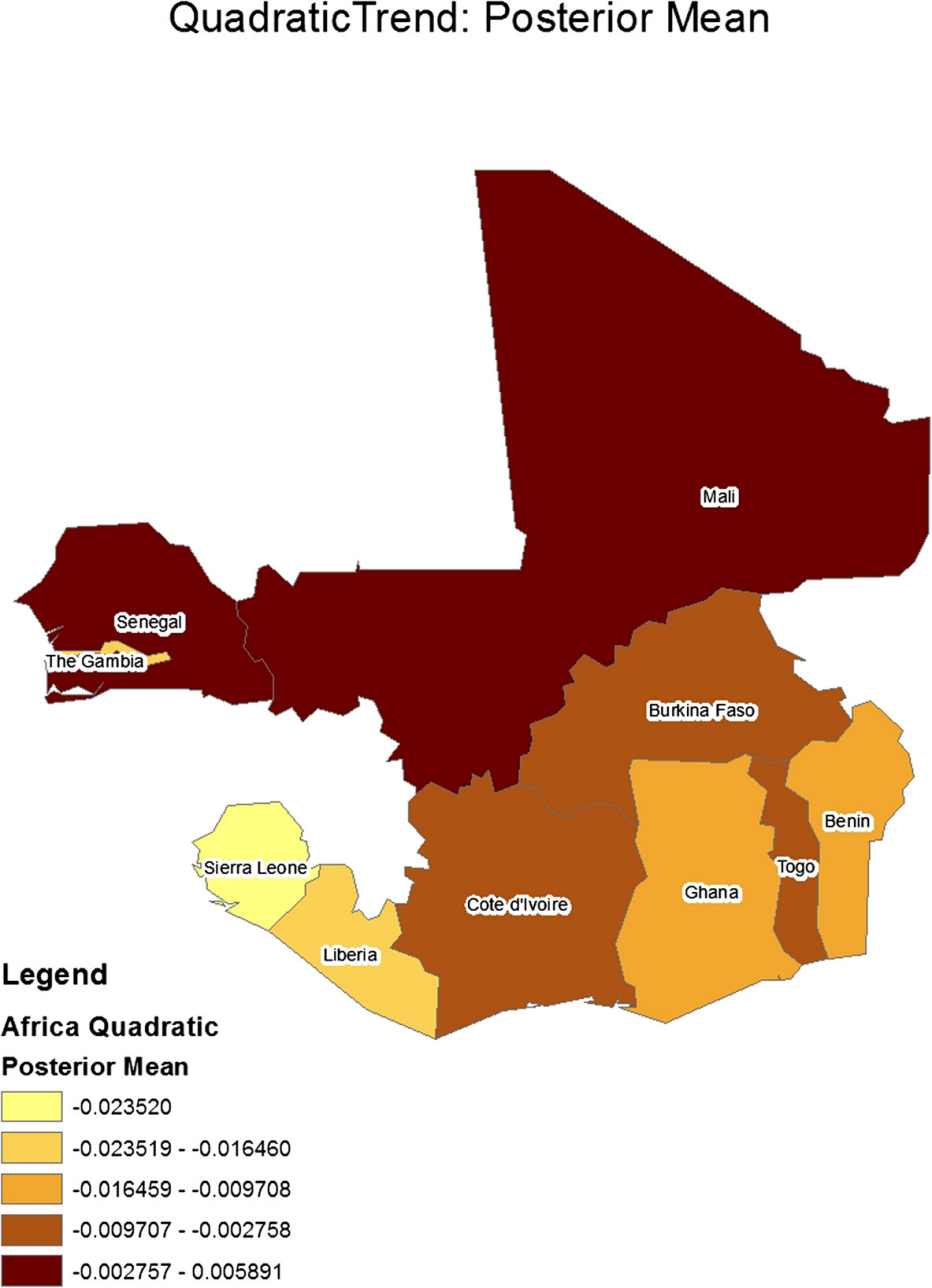
Geographical map of posterior mean results for the spatially-varying quadratic trend coefficient.

**Figure 6:**
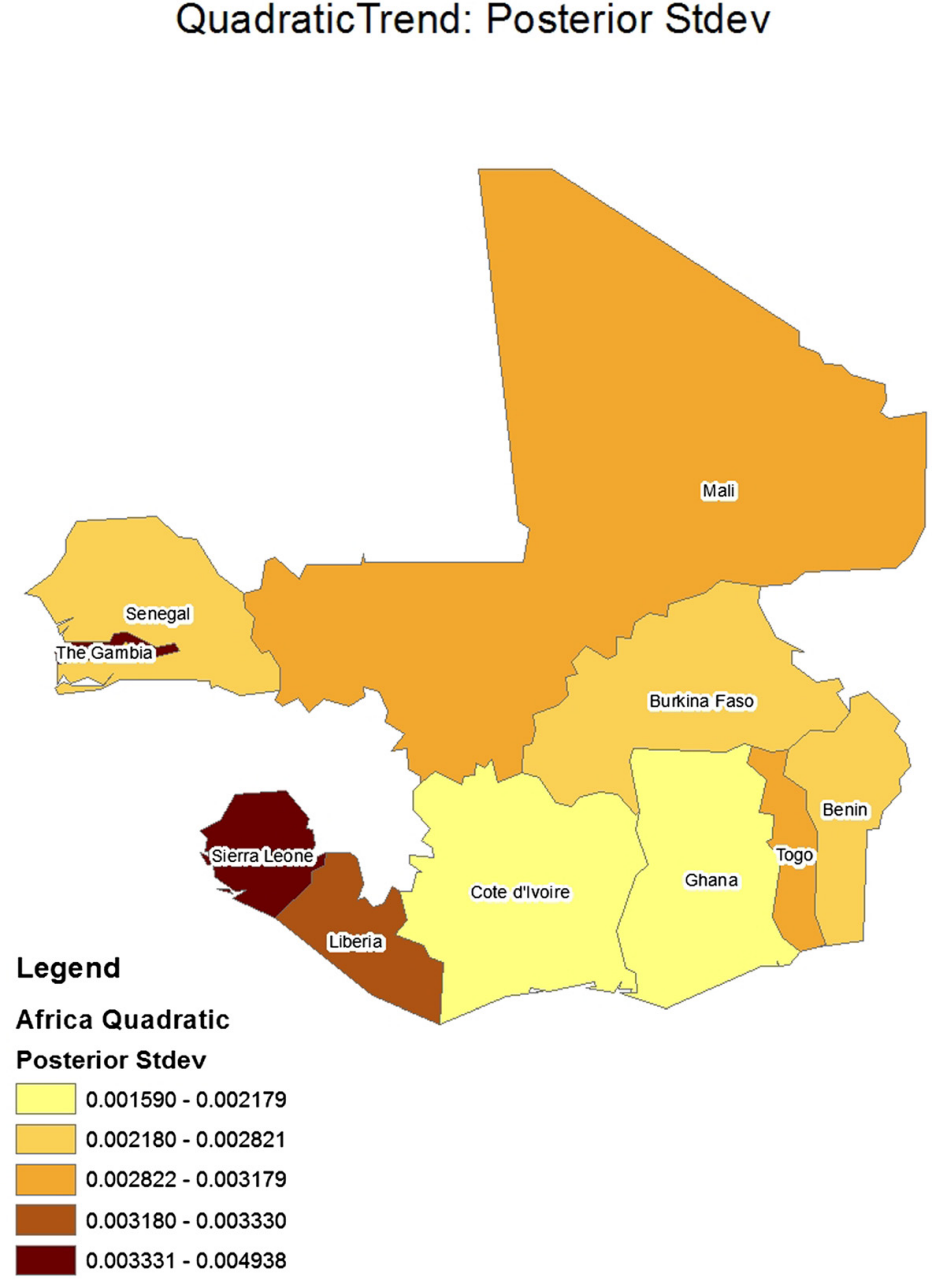
Geographical map of posterior standard deviation results for the spatially- varying quadratic trend coefficient.

Table 2 gives the constant regression coefficients for the model. Figures 3 and 4 show geographical maps of posterior results (mean and standard deviation, respectively) for the spatially varying linear trend term, ***μ***_1_ (i e, country-specific linear trend coefficients). Similarly, Figures 5 and 6 show geographical maps of posterior results (mean and standard deviation, respectively) for the spatially varying quadratic trend term, ***μ***_2_ (ie, country-specific quadratic trend coefficients).

**Table 2:** Posterior results for the constant regression coefficients

	**Mean**	**Sd**	**95% CI**
Intercept	-2.728	0.0444	(-2.814, -2.642)
Mstpr	0.0869	0.041	(0.0067, 0.1674)
Msepr	-0.114	0.0184	(-0.149, -0.0768)
Mtmp	-0.2034	0.02514	(-0.2528, -0.1545)
Dtpav	0.0615	0.0407	(-0.0184, 0.1416)
Mvp	0.1544	0.0432	(0.0705, 0.2402)
Dp	-0.1162	0.0203	(-0.1556, -0.0761)
Totp	-0.1055	0.0377	(-0.1808, -0.0325)

(columns from left to right: mean posterior, posterior standard deviation, 95% credible interval) for Model 1 (M1).

Most of the predictor variables are statistically significant (all except “dtpave”). In particular, as shown in Table 2, mean sea-level pressure (msepr), mean temperature (mtmp), number of days with precipitation ≥1 mm (dp) and total precipitation (totp) show negative association with malaria rate, ie, a decrease in either of these variables is associated with an increase in malaria rate. Table 3 shows same regression results as Table 2 but without the spatially varying trend term (i.e. trend term is constant). By comparing these results one can see the effect of including the spatially varying trend terms on the other regression coefficients. Mainly, mean station pressure (mstpr) becomes positively significant, and total precipitation (totp) becomes negatively significant. Therefore, a model that does not account for the spatially varying trends may miss important associations (such as the mean station pressure).

**Table 3:** Posterior results for the constant regression coefficients

	**Mean**	**Sd**	**95% CI**
Intercept	-2.537	0.025	(-2.586, -2.489)
Mstpr	0.032	0.033	(-0.033, 0.097)
Msepr	-0.1401	0.0177	(-0.174, -0.1043)
Mtmp	-0.1685	0.0193	(-0.2055, -0.1302)
Dtpav	0.0505	0.0345	(-0.017, 0.1187)
Mvp	0.298	0.0299	(0.24, 0.357)
Dp	-0.159	0.0176	(-0.194, -0.125)
Totp	-0.053	0.0284	(-0.1095, 0.002)

(columns from left to right: mean posterior, posterior standard deviation, 95% credible interval) for Model 3 (M3; ie, constant trend term).

Some limitations exist for this study. First, the United Nations defines 15 countries for West Africa; however, in this study only ten were considered due to the sparseness of the data for the other five countries. Also, the study uses annual and country averages as potential variables and thus may miss potentially significant seasonal and local-scale effects.

The main findings may shed light on better understanding the association between weather and climate variables and spread of malaria. Critically, the results show statistically significant association between malaria rates and several climate variables (temperature, precipitation and pressure) after accounting for spatiotemporal variability in the data. In most other studies of the impact of climate on malaria distribution, spatial and temporal variability in the data are ignored and data are modelled based on an incorrect assumption of independence. This sensitivity of malaria incidence to climate factors in Africa is generally consistent with literature [3,7,4,11]. The negative correlation between temperature and malaria rate could be explained by the no-linear response of malaria spread to temperature, with a negative association for higher temperatures. Note that mean annual temperatures in our study range between 26°C and 28°C, and it is possible that seasonal temperatures higher than 28°C might have occurred that, according to [8] are associated with decreased malaria rates. The study also found negative correlation between the total amount of annual rainfall and malaria rate, which is inconsistent with [7] and [11]. Compared to temperature, rainfall associations with malaria are less understood. In addition, rainfall is spatially and temporally much more variable than temperature and perhaps total annual precipitation may not be a good proxy for the seasonal values associated with malaria occurrence.

## Conclusions

Studies on the modelling of malaria incidence have shown that its relationship with environmental and socio-economic variables is inherently complex and spatially and temporally heterogeneous. This study introduces a hierarchical Bayesian modelling framework for the analysis of malaria distribution and its relationships with climate factors in ten West African countries. The proposed hierarchical model takes into account spatiotemporal dependencies through spatially varying linear and quadratic trend terms. The reported malaria cases and deaths for an 11-year period (1996-2006) were linked with annual climate data for each country. The two most important climate factors were found to be average annual temperature and total annual precipitation, and they show negative associations with malaria incidence.

A model without the spatially varying trend showed a positive association with total precipitation and no statistical significance for mean station pressure. Therefore, a model that does not account for the spatially varying term may misinterpret or totally miss important associations.

The proposed modelling approach appropriately accounts for spatial and temporal dependence typical in studies of infectious diseases such as malaria. Results demonstrate that the proposed modelling approach is robust and can be useful in understanding the impact of climate change on the spread of malaria. Additionally, the model can be applied to analyse the spread of other infectious diseases and in optimizing management efforts (eg, drug policy changes) on the spread of malaria. With a more rigorous effort, this modelling framework can be extended to account for socio-economic factors as well as other important factors such as access to health, information on drug policy, and drug resistance.

## Abbreviations

DIC: Deviance information criterion; AIC: Akaike's information criterion; MCMC: Markov chain Monte Carlo; NOAA: National Oceanic and Atmospheric Administration; NCDC: National Climate Data Center.

## Competing interests

The authors declare that they have no competing interests.

## Authors’ contributions

AA carried out the statistical modelling and drafted the manuscript. MCJ carried out the data collection and mapping. CK carried out the literature review, drafted the manuscript and carried out preliminary data analysis. All authors read and approved the final manuscript.
